# Vasculitis, cerebral infarction and persistent *Bartonella henselae* infection in a child

**DOI:** 10.1186/s13071-016-1547-9

**Published:** 2016-05-10

**Authors:** Nandhakumar Balakrishnan, Marna Ericson, Ricardo Maggi, Edward B. Breitschwerdt

**Affiliations:** Department of Clinical Sciences and the Intracellular Pathogens Research Laboratory, Center for Comparative Medicine and Translational Research, College of Veterinary Medicine, North Carolina State University, Raleigh, NC USA; Cutaneous Imaging Center, Department of Dermatology, University of Minnesota, Minnesota, USA

**Keywords:** Neurobartonellosis, *Bartonella henselae* San Antonio 2 vasculitis

## Abstract

**Background:**

The genus *Bartonella* is comprised of a rapidly increasing number of pathogenic species that induce a seemingly diverse spectrum of neurological symptoms. During the 12 year period that followed the initial onset of neurological and gastrointestinal symptoms, an 11 year-old girl experienced a spectrum of neurological complaints including frequent headaches, visual and auditory hallucinations, anxiety, vision loss involving the lower left quadrant of both eyes, episodic bouts of generalized paralysis, facial palsy, chronic insomnia, seizures, dizziness, cognitive dysfunction, and memory loss. PCR assays targeting *Bartonella* spp. were used to test formalin-fixed, paraffin embedded brain tissue, patient blood specimens and Bartonella alpha Proteobacteria growth medium (BAPGM) enrichment blood cultures. PCR positive amplicons were sequenced directly and compared to GenBank sequences. *Bartonella* spp. serology was performed by indirect fluorescent antibody testing and confocal laser scanning microscopy was used to visualize *B. henselae* organisms in resected brain.

**Results:**

*Bartonella henselae* DNA was independently PCR amplified and sequenced from the girl’s right parietal lobe, surgically resected in 2000 and from a blood specimen collected in 2012. Although causation cannot be established by a case report, prior diagnostic testing resulted in findings that were either inconclusive or within normal reference ranges and no etiological diagnosis had been obtained to explain the patient’s initial or progressive neurological symptoms.

**Conclusions:**

As intravascular, intra-erythrocytic and endotheliotropic bacteria, it is possible that *B. henselae* initially induced a vasculitis, resulting in secondary cerebral infarction, tissue necrosis and surgical resection. *Bartonella* bacteremia, potentially spanning a 12-year time frame, in conjunction with the therapeutic administration of immunosuppressive drugs may have resulted in a progression and potentiation of the neurological disease that was partially reversible following antibiotic administration.

## Background

As recently reviewed, an increasing number of *Bartonella* species have been identified as zoonotic pathogens that are transmitted by animal bites or scratches, needle sticks, blood transfusions, or by arthropods [1–4]. Potentially, because *Bartonella* spp. can infect erythrocytes, endothelial cells, pericytes, and various macrophage-type cells, vascular pathology may be much more diverse than is currently appreciated [5–7]. Due to current limitations associated with diagnostic testing for bartonellosis, a high index of suspicion is required, particularly in patients with occult, persistent bacteremia, small vessel disease, or non-specific symptoms, such as fatigue, insomnia and memory loss [6, 8, 9]. Because of the rapid discovery of new, pathogenic *Bartonella* spp., the expanding number of arthropods proven or suspected in transmission, the large numbers of infected animal reservoir hosts in nature, and the broad spectrum of neurological abnormalities reported in recent years, neurobartonellosis may be a much more prevalent disease in both immunocompetent and immunocompromised patients throughout the world than is currently recognized [6].

## Case report

In March 2000, an 11-year-old girl residing in Ottawa, Canada developed sudden-onset, headaches, difficulty walking, left sided paresis and an ataxic gait. The family resided in a rural environment and their home backed onto an extensive ravine. Shortly before the onset of neurological symptoms, a feral dog was adopted from the local humane society. Historically, the owner did not observe fleas or ticks and the dog did not bite the child. However, shortly after adoption, the dog developed a large abscess with purulent discharge in the neck region, which the child cleaned daily. A few weeks later, the girl developed flu-like symptoms followed by progressive neurological abnormalities. Concurrently, she reported gastrointestinal symptoms, including abdominal pain, bloating and constipation, which persisted throughout the patient’s subsequent illness. A Magnetic resonance imaging (MRI) scan identified a large, focal, demyelinating mass lesion located in the right parietal lobe. Based upon the MRI and examination of frozen brain tissue sections obtained at surgery, the mass was presumptively diagnosed by a neuropathologist as a glioblastoma. The mass and a portion of the right parietal lobe were surgically resected. Based upon formalin-fixed paraffin embedded (FFPE) tissue histopathology, the diagnosis was revised to a reactive inflammatory process consistent with vasculitis, secondary cerebral infarction, and tissue necrosis. The histopathological examination revealed abrupt demarcations between necrotic areas, with accompanying destruction of axons and myelin. Adjacent brain tissue contained wide-spread perivascular lymphoplasmacytic infiltration with extension into the vessel wall, resulting in intimal proliferation and sparse hemosiderin deposition in both venules and small arteries. There was no evidence of selective perivascular demyelination. The majority of the perivascular lymphocytes were T cells, with a few scattered B cells. Mindbomb E3 ubiquitin protein ligase 1(MIB1) staining was minimal, except among lymphocytes, and the tissue was immunonegative for Epstein-Barr virus (EBV). During the next 3 years, the patient was intermittently treated for a presumptive autoimmune neurological disease with high dose intravenous corticosteroids. Her diagnoses included idiopathic vasculitis, Guillain-Barre syndrome, multiple sclerosis, and acute disseminated encephalomyelitis (ADEM).

In 2003, the patient experienced frequent headaches, chest pain, visual and auditory hallucinations, anxiety, ocular floaters, severe depression, and fatigue. Additional episodes of partial paralysis occurred in 2004 and 2009. A few weeks prior to each of the three episodes, the patient experienced a non-febrile respiratory illness. In July 2004, after returning home from a sailing camp, the girl developed neurocognitive abnormalities, after which there was rapid deterioration in muscle strength with left sided paralysis, homonymous hemianopia, seizures, dysphagia, laryngitis, and severe confusion. After initiation of intravenous immunoglobulin (IVIG) the patient slowly recovered; however, it was several months before she became ambulatory, and her gait never normalized. During this time, the patient reportedly developed severe allergic reactions when ingesting gluten, corn, lactose or pork.

In March 2009, the patient again developed progressive paresis that was treated with low dose IVIG for 3 days. Despite initial improvement in neurological status, she developed generalized seizures and status epilepticus a few days later, requiring plasma exchange and pharmacological-induction of a coma, lasting for 10 days. After awakening from the coma, the patient exhibited tetraplegia, dysphasia, and marked facial palsy. During hospitalization, treatment consisted of IVIG and fluconazole (Diflucan) for oral Candidiasis. Serum IgA, IgM and IgG levels were consistently low, and based upon repeated testing had never normalized. Due to her unusual clinical presentation and disease progression, a diagnosis of tumefactive multiple sclerosis was rendered at this time. Shortly following this hospitalization, the family physician suspected Lyme disease, with equivocal Western immunoblot results (IgM positive, IgG negative), the patient was treated with penicillin. Combination therapy with fluconazole, penicillin and IVIG appeared to facilitate rapid improvement in neurological status, including improved ambulation, decreased headaches, and improved cognitive function.

Overall, there was a cumulative deterioration in neurological status following each of the three distinct paralysis episodes (2000, 2004, 2009); however, there was no observable disease progression between these episodes. After 2009, the patient’s neurological disabilities resulted in limited daily activities and required physical support and full-time care. In October of 2011, based upon the patient’s historical symptoms, a physician in Vancouver clinically suspected co-infections consisting of Lyme disease, babesiosis and neurobartonellosis. Because of a history of night sweats (starting in 2000), the patient was treated with atovaquone (Mepron) 1 tsp. twice daily, azithromycin (Zithromax) 500 mg, intravenously once daily and ceftriaxone (Rocephin®) 2 g intravenously 5 days each week for 8 weeks. After initiation of this treatment regimen, the patient experienced dramatic improvement, including partial resolution of facial palsy within 2 weeks, resolution of severe jaw and chest pain, cessation of night sweats, clearer thinking and speech, and a decrease in the frequency of floaters; all historical problems reported since illness onset. On February 14, 2012, the mother contacted the corresponding author by email and requested that her daughter be entered into an Institutional Review Board (North Carolina State University, College of Veterinary Medicine, IRB #164-08-05, NCSU-CVM IRB) approved research study.

## Methods

### Serology

For this study, all serum samples were tested by indirect fluorescent antibody (IFA) assays using a panel of *Bartonella* antigens. Briefly, antibody responses to *Bartonella henselae* strain Houston I, *B. henselae* strain San Antonio 2 (SA2 strain type), *B. vinsonii* subspecies *berkhoffii* genotype I, *B. vinsonii* subspecies *berkhoffii* genotype II*, B. vinsonii* subspecies *berkhoffii* genotype III, and *B. koehlerae* were tested by IFA as previously described [4–6]. Seropositive samples were defined as having endpoint titers ≥1:64 using a twofold dilution scale of 1:16 – 1:8192.

### Molecular testing

*Bartonella* testing was performed using the *Bartonella* alpha proteobacteria growth medium (BAPGM) platform, as previously described [4–8]. The primer sequences targeting *Bartonella* genus specific 16-23S intergenic transcribed spacer (ITS) and *B. koehlerae* species specific 16-23S ITS were used [4–8]. BAPGM cultures were processed in a biosafety cabinet with HEPA filtration in a limited access Biosafety Level II laboratory. To assess for potential contamination during blood sample processing into BAPGM, an un-inoculated BAPGM culture flask was processed simultaneously and in an identical manner with each batch of patient blood and serum samples tested.

DNA extraction from formalin fixed paraffin-embedded (FFPE) brain tissues were tested by PCR for the presence of *Bartonella* spp. DNA. Multiple 30μm sections of FFPE brain tissue was excised using a microtome. Previously, we described *Bartonella* spp. DNA carryover, during animal necropsy and during the subsequent processing of tissue samples [10]. Hence, special precautions were taken in our laboratory when sampling paraffin blocks to minimize the DNA cross contamination. A negative control paraffin block, containing no tissue, was cut in between each tissue block and processed in an identical manner as samples to determine if any DNA carryover was occurring through the use of the microtome. Tissues were processed in small batches and the work surface was thoroughly cleaned using ethanol and DNAse between each tissue block tested, to avoid *Bartonella* spp. DNA carry over between the samples. DNA was extracted using QIAamp FFPE Tissue Kit (Qiagen, Valencia, CA) following manufacturer’s instructions. Elution buffer was used as a reagent control with each set of DNA extractions. DNA concentrations and purity were determined using a spectrophotometer (Nanodrop, Wilmington, DE). Extracted DNA was stored at -20 °C.

### Laser confocal immunohistochemistry

To visualize *B. henselae,* brain tissue sections were processed using immunostaining as described previously [11]. Briefly, 20μm paraffin-embedded brain tissue sections were incubated with monoclonal *Bartonella henselae* antibody (Abcam Cambridge, MA) at 1:100 dilution followed by donkey anti-mouse IgG conjugated to Alexa Fluor 594 (Jackson ImmunoResearch West Grove, PA) at 1:400 dilution. Secondary antibody control includes buffer and donkey anti-mouse IgG conjugated to Alexa Fluor 594 (1:400 dilution) only.

## Results and discussion

**Fig. 1:**
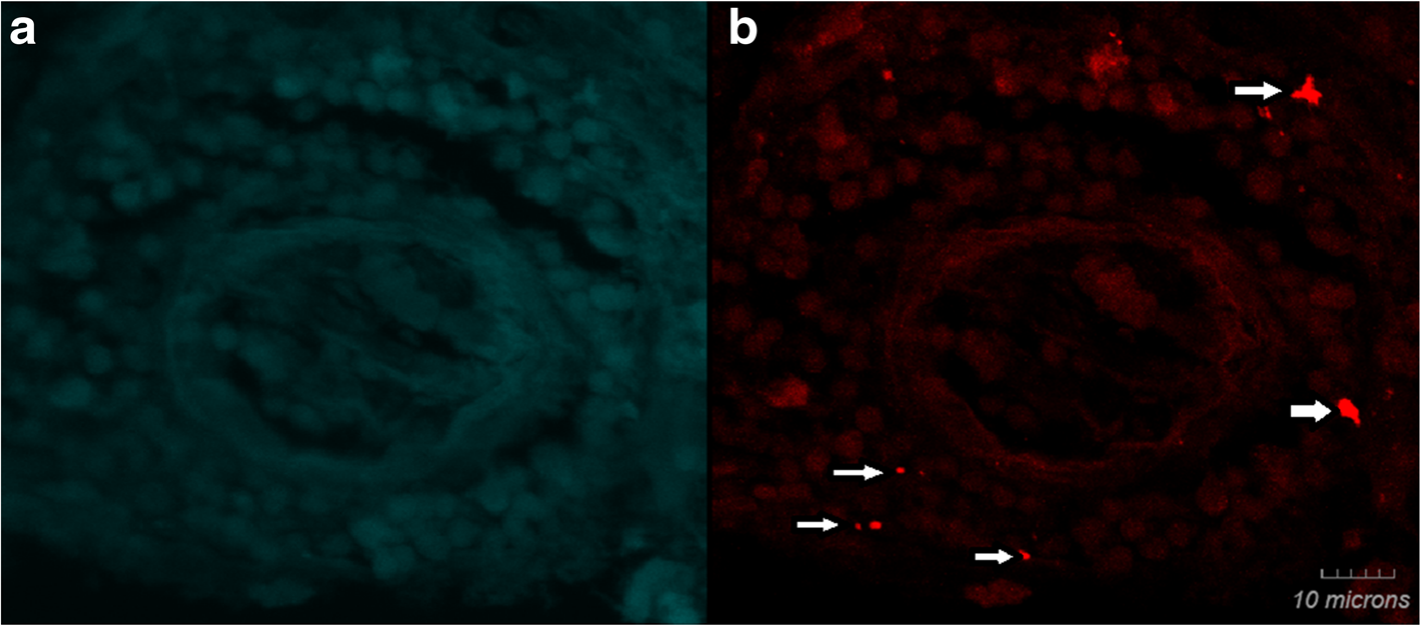
*Bartonella henselae* immunoreactivity detected in brain tissue biopsy using laser scanning confocal microscopy. Panel **a** shows no immunoreactivity (secondary antibody control). Panel **b** (arrows) shows immunoreactive *B. henselae*. Stained samples were imaged using laser scanning confocal microscopy. Z-stack projections for both images are in-focus projections of 17 0.43μm optical sections for a total thickness of 7 μm. (Olympus PlanApo 63×/1.40 oil objective)

By IFA testing using previously described antigens and techniques, the patient was not seroreactive to *B. henselae* (Houston 1 strain type), *B. henselae* (SA2 strain type), *B.vinsonii* subsp. *berkhoffii* genotypes I, II and III and *B. koehlerae.* Using a triple draw approach [12] and previously described PCR assays targeting *Bartonella* genus specific 16-23S ITS and *B. koehlerae* species specific 16-23S ITS elements, *B. henselae* (376/380 base pairs, 99 %) DNA was amplified and sequenced from one of three of the patient’s blood specimens. At the time of sample collection, the patient was being treated with azithromycin and ceftriaxone. BAPGM enrichment blood cultures were PCR negative following 7 and 14 day incubation periods and no subculture isolate was obtained. Subsequently, *B. henselae* (448/448 base pairs, 100 %) was amplified from the surgically obtained, April 2000 FFPE brain tissue. Intracellular localization of *B. henselae* in surgically resected FFPE brain tissue was visualized by confocal laser scanning microscopy (Fig. 1).

When BAPGM enrichment PCR testing was performed in March, 2012, the girl was still experiencing severe cognitive problems, essential tremors and had musculoskeletal pain and weakness. Based on amplification of *B. henselae* from blood and brain tissue, her family physician initiated antibiotic treatment with intravenous ceftriaxone (2g intravenously daily), metronidazole (Flagyl, 250 mg twice daily), and azithromycin (500 mg daily) for 9 weeks. During the first 2 weeks of therapy, the patient experienced worsening symptoms, including cognitive decline, seizures, generalized muscle and joint pain, after which there was gradual improvement in overall cognitive function. By August 2012, muscle tone, visual stress and memory had improved dramatically. By October 2012, the patient’s mother reported continued improvement in neurological status. Subsequently, the patient developed a flu-like illness; however, unlike the three previous respiratory episodes and for the first time in 12 years she developed a fever (104 °F), and did not subsequently develop paralysis.

In October 2012, the patient was retested in conjunction with the NCSU-CVM IRB study. Prior to sample collection, the patient was not receiving antibiotics. Based upon repeated IFA testing, she was not seroreactive against *B. henselae* (Houston 1 strain type) *B. henselae* (SA2 strain type), *B. vinsonii* subsp. *berkhoffii* genotypes I, II and III and *B. koehlerae* antigens*.* Using the triple draw approach (3 samples sets pulled every other day) [12], *B. henselae* DNA was not amplified from blood, serum, and post-enrichment 7 and 14 day BAPGM enrichment blood cultures. No subculture isolates were obtained. Due to the chronicity and complexity of the patient’s illness and because based upon prior instances of rapid neurological deterioration, she refused additional treatment with immunosuppressive drugs. On follow up, facial palsy has resolved, neurocognitive function and physical movement have marginally improved, and her thought processes, (i.e. memory and ability to plan and follow complex operations) have remained stable. MRIs performed in 2015 and 2016 showed frontal lobe disintegration without evidence of progressive pathology in other portions of the brain.

Since 2000, when this patient was initially examined, there has been a substantial increase in the body of clinical literature describing a spectrum of symptoms in patients infected with *B. henselae.* Despite progress, *Bartonella* species in general, and *B. henselae* in particular, remain incompletely understood in the context of cellular and vascular tropism, neuropathogenesis, and the role that persistent intravascular infection plays in disease expression [6]. During the 12 year period following the initial onset of neurological and gastrointestinal symptoms, this patient experienced a wide spectrum of neurological abnormalities including frequent headaches, visual and auditory hallucinations, anxiety, vision loss involving the lower left quadrant of both eyes, episodic bouts of generalized paralysis, facial palsy, chronic insomnia, seizures, dizziness, cognitive dysfunction, and memory loss. During this time frame, differential neurological diagnoses included gliobastoma (initial mass lesion), vasculitis of undetermined etiology, Guillain-Barre syndrome, acute disseminated encephalomyelitis (ADEM), and multiple sclerosis. Based upon retrospective testing of the parietal lobe mass lesion that was surgically resected in 2000, a diagnosis of neurobartonellosis seems justified in this patient. Based upon DNA sequence comparison, *B. henselae* was successfully PCR amplified from the FFPE brain tissue and from a blood specimen obtained in 2012. Using a previously described technique [11], *B. henselae* organisms could be visualized in FFPE surgical brain tissue by laser scanning confocal microscopy. Additional indirect support for a diagnosis of neurobartonellosis was provided by the historical deterioration in neurological status that followed administration of immune suppressive drugs, as compared to gradual improvement in neurological status after the initiation of antimicrobial therapy.

The extent to which repeated, corticosteroid-induced suppression of immune function contributed to additional or progressive neurological damage is unknown. However, there are case reports in which patients treated with immunosuppressive drugs based upon a “positive” autoimmune disease test result subsequently developed *B. henselae* endocarditis [13, 14]. There are also recent case reports that describe the medical complexities associated with differentiating occult, intravascular infection with a *Bartonella* sp. from autoimmune diseases [15]. The extent to which repeated administration of IVIG prevents or enhances disease progression in patients with occult infection also deserves critical consideration. IVIG administration to a child with Guillain-Barre syndrome did not halt the progressive paralysis prior to an apparent reversal in the disease process after initiation of antibiotic therapy [16].

It is also unclear whether persistent intravascular, microglial, or cerebrospinal fluid (CSF) infection contributed to the various neurological symptoms or to the three paresis/paralysis episodes experienced by this patient between 2000 and 2009. Based upon in vitro and in vivo evidence, *B. henselae* is an intraerythrocytic and endotheliotropic bacteria [17]. However, no studies have addressed the potential localization of the bacterium within the vasculature versus brain parenchymal tissues or CSF of human patients. Unfortunately, despite substantial efforts on the part of several research groups, a pathologically relevant rodent model for bartonellosis or neurobartonellosis has not been established. Thus, prospective patient studies are needed to document intravascular and/or CSF infection with *B. henselae,* in conjunction with direct therapies to eliminate the infection and determine subsequent patient outcomes.

Combined utilization of molecular-based diagnostic testing, in conjunction with conventional microbiological approaches, are increasingly used in the clinical setting for the detection of fastidious and non-fastidious pathogens. While the location of *Bartonella* during the nonbacteremic phase of infection is currently unknown, endothelial cells and bone marrow have been hypothesized as primary niches in both incidental and reservoir hosts [18, 19]. Negative BAPGM enrichment blood culture is often attributed to history of antibiotic treatment before sampling as reported in this patient. A cross-sectional study by Maggi et al. [20] found that 75 % of *Bartonella*-infected humans did not have IFA antibodies to the infecting *Bartonella* species or genotype. Although unproven, chronic intravascular infection with *Bartonella* spp. may induce a degree of immunological anergy, resulting in an undetectable level of organism-specific antibodies in naturally-infected human patients or other mechanisms may contribute to seronegativity.

The source of *B. henselae* infection was not established for this patient. Although cats are most often implicated in the transmission of *B. henselae* to humans (Cat Scratch Disease), dogs have been infrequently reported as a source of infection. Shortly before the onset of a flu-like illness, this child did participate in cleaning purulent material from an abscess in the dog’s neck region, which could have potentially contained viable bacteria. *Bartonella henselae* can be isolated from the lymph nodes of dogs and humans with granulomatous lymphadenitis [21] Recently, French investigators implicated *B. henselae* and three *Bartonella* spp. not previously isolated from humans, as a cause of undifferentiated chronic illness, potentially transmitted by ticks [22]. The numerous modes of transmission in conjunction with the increasingly large number of animals in which *B. henselae* bacteremia has been confirmed supports a more overarching disease designation (bartonellosis), rather than the historical designation of *B. henselae-*induced Cat Scratch Disease.

## Conclusion

If *B. henselae* contributed to the onset and progression of this patient’s illness, a delay in diagnosis and administration of immunosuppressive drugs may have resulted in a progression and potentiation of the neurological disease, which might have been more effectively managed pre-surgically by directed antimicrobial therapy. In a 2004 *Nature* publication by Merrell & Falkow [23], *Helicobacter pylori* and *B. henselae* were used to exemplify the role of “stealth pathogens” in chronic disease causation. Stealth pathogens are characterized by slow bacterial replication time (approximately 22h for *B. henselae*), a long incubation period (potentially months to years), induction of chronic symptomatology, a non-sterilizing immune response, indirect modes of transmission (all *Bartonella* spp. are known or thought to be transmitted by one or more arthropod vector), and a persistently infected “carrier state”. Based upon evolving evidence, *B. henselae* appears to fulfill all criteria for a stealth, intravascular, and endotheliotropic pathogen that can induce a chronic neurological symptoms.

## Consent

Written informed consent was obtained from the patient for publication of this report and the accompanying images.

## References

[1] Chomel BB, Boulouis HJ, Breitschwerdt EB, Kasten RW, Vayssier-Taussat M, Birtles RJ (2009). Ecological fitness and strategies of adaptation of *Bartonella* species to their hosts and vectors. Vet Res.

[2] Pulliainen AT, Dehio C (2012). Persistence of *Bartonella* spp. stealth pathogens: from subclinical infections to vasoproliferative tumor formation. FEMS Microbiol Rev.

[3] Ben-Tekaya H, Gorvel J-P, Dehio C (2013). *Bartonella* and *Brucella*- weapons and strategies for stealth attack. Cold Spring Harb Perspect Med.

[4] Breitschwerdt EB (2014). Bartonellosis: one health perspectives for an emerging infectious disease. ILAR J.

[5] Breitschwerdt EB, Linder KL, Day MJ, Maggi RG, Chomel BB, Kempf VAJ (2013). Koch’s postulates and the pathogenesis of comparative infectious disease causation associated with *Bartonella* species. J Comp Pathol.

[6] Breitschwerdt EB, Sontakke S, Hopkins S (2012). Neurological manifestations of Bartonellosis in immunocompetent patients: a composite of reports from 2005–2012. Journal of Neuroparasitology.

[7] Breitschwerdt EB, Kordick DL, Gavins FNE, Stokes KY (2016). *Bartonella* species and vascular pathology (Chapter 6). Vascular response to pathogens.

[8] Breitschwerdt EB, Maggi RG, Nicholson WL, Cherry NA, Woods CW (2008). *Bartonella* sp. bacteremia in patients with neurological and neurocognitive dysfunction. J Clin Microbiol.

[9] Maggi RG, Mozayeni BR, Pultorak EL, Hegarty BC, Bradley JM, Correa M (2012). *Bartonella* spp. bacteremia and rheumatic symptoms in patients from Lyme disease-endemic region. Emerg Infect Dis.

[10] Varanat M, Maggi RG, Linder KE, Horton S, Breitschwerdt EB (2009). Cross-contamination in the molecular detection of *Bartonella* from paraffin-embedded tissues. Vet Pathol.

[11] Maggi RG, Ericson M, Mascarelli PE, Bradley JM, Breitschwerdt EB (2013). *Bartonella henselae* bacteremia in a mother and son potentially associated with tick exposure. Parasit Vectors.

[12] Pultorak EL, Maggi RG, Mascarelli PE, Breitschwerdt EB (2013). Serial testing from a 3-day collection period by use of the *Bartonella* Alphaproteobacteria growth medium platform may enhance the sensitivity of *Bartonella* species detection in bacteremic human patients. J Clin Microbiol.

[13] Turner JW, Pien BC, Ardoin SA, Anderson AM, Shieh WJ, Zaki SR (2005). A man with chest pain and glomerulonephritis. Lancet.

[14] Vikram HR, Bacani AK, DeValeria PA, Cunningham SA, Cockerill FR (2007). Bivalvular *Bartonella henselae* prosthetic valve endocarditis. J Clin Microbiol.

[15] Maritsi DN, Zarganis D, Metaxa Z, Papaioannou G, Vartzelis G. *Bartonella henselae* infection: an uncommon mimicker of autoimmune disease. Case Rep Pediatr. 2013;1–4. 10.1155/2013/726826 PMC3562603 23424700

[16] Mascarelli PE, Maggi RG, Hopkins S, Mozayeni BR, Trull CL, Bradley JM (2013). *Bartonella henselae* infection in a family experiencing neurological and neurocognitive abnormalities after woodlouse hunter spider bites. Parasit Vectors.

[17] Harms A, Dehio C (2012). Intruders below the radar: molecular pathogenesis of Bartonella spp. Clin Microbiol Rev.

[18] Mändle T, Einsele H, Schaller M, Neumann D, Vogel W, Autenrieth IB (2005). Infection of human CD34^+^ progenitor cells with *Bartonella henselae* results in intraerythrocytic presence of *B. henselae*. Blood.

[19] Balakrishnan N, Cherry NA, Linder KE, Pierce E, Sontakke N, Hegarty BC (2013). Experimental infection of dogs with *Bartonella henselae* and *Bartonella vinsonii* subsp. *berkhoffii*. Vet Immunol Immunopathol.

[20] Maggi RG, Mascarelli PE, Pultorak EL, Hegarty BC, Bradley JM, Mozayeni BR (2011). *Bartonella* spp. bacteremia in high-risk immunocompetent patients. Diagn Microbiol Infect Dis.

[21] Duncan AW, Marr HS, Birkenheuer AJ, Maggi RG, Williams LE, Correa MT (2008). *Bartonella* DNA in the blood and lymph nodes of Golden Retrievers with lymphoma and in healthy controls. J Vet Intern Med.

[22] Vayssier-Taussat M, Moutailler S, Féménia F, Raymond P, Croce O, La Scola B (2016). Identification of novel zoonotic activity of *Bartonella* spp., France. Emerg Infect Dis.

[23] Merrell DS, Falkow S (2004). Frontal and stealth attack strategies in microbial pathogenesis. Nature.

